# Road to Better Work-Life Balance? Lean Redesigns and Daily Work Time among Primary Care Physicians

**DOI:** 10.1007/s11606-021-07178-6

**Published:** 2021-12-09

**Authors:** Dorothy Y. Hung, Gabriela Mujal, Anqi Jin, Su-Ying Liang

**Affiliations:** 1 School of Public Health, University of California at Berkeley, Berkeley, CA USA; 2 Sutter Health, Palo Alto Medical Foundation Research Institute, Palo Alto, CA USA

**Keywords:** primary care redesign, lean management, work efficiency, physician work time, time-stamped EHR access logs, interrupted time series analysis, longitudinal data

## Abstract

**Purpose:**

To assess the impact of Lean primary care redesigns on the amount of time that physicians spent working each day.

**Methods:**

This observational study was based on 92 million time-stamped Epic® EHR access logs captured among 317 primary care physicians in a large ambulatory care delivery system. Seventeen clinic facilities housing 46 primary care departments were included for study. We conducted interrupted time series analysis to monitor changes in physician work patterns over 6 years. Key measures included total daily work time; time spent on “desktop medicine” outside the exam room; time spent with patients during office visits; time still working after clinic, i.e., after seeing the last patient each day; and remote work time.

**Results:**

The amount of time that physicians spent on desktop EHR activities throughout the day, including after clinic hours, decreased by 10.9% (95% CI: −22.2, −2.03) and 8.3% (95% CI: −13.8, −2.12), respectively, during the first year of Lean implementation. Total daily work hours among physicians, which included both desktop activity and time in office visits, decreased by 20% (95% CI: −29.2, −9.60) by the third year of Lean implementation.

**Conclusions:**

These findings suggest that Lean redesign may be associated with time savings for primary care physicians. However, since this was an observational analysis, further study is warranted (e.g., randomized trial) —to determine the impact of Lean interventions on physician work experiences.

## INTRODUCTION

Health systems are seeking ways to improve efficiency in primary care where high patient demand and physician burnout continue to present challenges among the workforce. According to one study, 44% of physicians report working more than 60 h per week with over one-third of physician time consumed by administrative tasks (e.g., billing, staffing, scheduling).^1,2^ Time-motion studies also provide in-depth examination of how physicians utilize daily work hours. Based on both direct observation and analysis of time-stamped electronic health record (EHR) event logs, these studies find that primary care physicians spend approximately 2 h on EHR desktop tasks for every 1 h of direct patient care, and nearly half the day working in the EHR on documentation, order entry, and coding requirements.^3,4^

Changing when, where, and how much time is spent on “desktop medicine” or administrative tasks can reduce physician workload while releasing time for more direct patient care. Such adjustments are needed as over half of primary care physicians report symptoms of burnout, including emotional exhaustion and a low sense of personal accomplishment in their medical practice.^5–8^ One potential solution involves the growing use of industry-based Lean techniques to optimize work processes in healthcare settings. Adapted from manufacturing, Lean is a set of organizational principles, practices, and problem-solving tools designed for improving quality and production processes.^9^ Lean redesigns aim to streamline workflows and balance task distributions, which may result in more appropriate allocation of time and effort that primary care physicians spend on various activities throughout the day.

Some defining features of a Lean system include standardizing work to improve efficiency, eliminating waste, and engaging staff in frontline improvement work.^10,11^ Based on prior research reporting on Lean benefits as well as unanticipated results in healthcare,^12–21^ we examine whether Lean primary care redesigns can support physician practices that would result in more efficient patient care and completion of EHR-related tasks. At the same time, unintended consequences may include decreased physician satisfaction or increased burnout as a result of the workflow changes.^22^ This is a particular concern among primary care physicians who continue to battle long-standing challenges of high patient demand, low capacity due to workforce shortages, and limited time for quality improvement initiatives.

To assess the effects of Lean primary care redesigns on physician practice patterns and time working each day, we utilized EHR data sourced from 6 years of time-stamped Epic® EHR access logs. Lean redesigns were implemented in consecutive phases over time in all primary care clinics at a large, ambulatory care delivery system.^15^ This implementation occurred using a standard sequence that included (1) “5S” (“Sort, Set in order, Shine, Standardize, Sustain”), used as a method for organizing and standardizing medical equipment and patient education materials in all exam rooms; (2) co-locating physician and medical assistant dyads in a shared workspace; and (3) streamlining care team workflows. Anticipated results included higher efficiency in delivering patient care and completing tasks outside the exam room, leading to time savings particularly at the end of each day. Using longitudinal EHR data, we re-constructed daily physician practice patterns before, during, and after clinic hours to determine key points of daily activity. This was followed by assessment of changes in the total amount of time that physicians spent working each day after implementing Lean ​primary care redesigns. 

## METHODS

### Data Source and Measures

Our observational study was based on 92 million Epic® EHR time-stamped access logs captured daily among 317 primary care physicians over 6 years. Physicians were located across 46 primary care departments (Internal Medicine, Family Medicine, Pediatrics) housed within 17 clinic facilities at a large ambulatory system serving nearly one million patients. Executive leaders introduced Lean as a strategic initiative beginning in primary care. This began as a proactive effort to address challenges in health care, including growing patient demand and increasing pressure to “do more with less,” a fundamental aim of Lean thinking.

With the support of Lean consultants and internal trainers consisting of local leaders and physician champions, frontline primary care providers and staff developed a set of Lean redesigns to address common pain points and areas in need of improvement. In some clinics across the system, slight reconfiguration of exam rooms and back-office space was needed to better accommodate Lean designs such as 5S or co-location of care teams. Improvements in both the organization of space and care team protocols aimed to increase workflow efficiency and time savings each day (e.g., less time spent searching for equipment, more timely communication among team members). Corresponding efforts to sustain changes included use of weekly “Flow metrics” measuring a series of EHR-documented task completion times.^23^ These metrics were reported at the physician level and posted on back-office display boards for use during care team huddles and tiered reporting with organizational leaders and executives.

Leveraging Epic® access logs, we created several measures of time when physicians were engaged in either “desktop” activities (e.g., EHR documentation, care management, administrative tasks) or direct patient care. These time measures included (1) total desktop work time, which excluded time spent with patients during office visits; (2) time working after clinic hours, i.e., after the last patient visit of the day; (3) total time in direct contact with patients during office visits; (4) time working remotely outside clinic facilities; and (5) total daily work time.

To minimize the effects of turnover, we conducted our analysis among 317 physicians who were continuously employed from 2011 to 2016, during which Lean was implemented in phases in all primary care clinics across the health system. Continuous employment during this period was defined as a minimum 5% FTE for at least half of the months both before and after Lean was implemented. Additionally, physicians were included for analysis if they were employed for at least 6 months during the pre-Lean period, and at least 12 months during the post-Lean period. The full observational study period captured a minimum 1 year of baseline data, and a minimum 2 years of post-Lean data in all primary care clinics.

### Statistical Analysis

We employed segmented regression with interrupted time series analysis^24–26^ to assess impacts of Lean redesigns on physician work patterns. Analyses were based on a non-randomized, quasi-stepped wedge design with one-way crossover^27,28^ reflecting the phased implementation of Lean in all primary care clinics across the system. We used generalized linear mixed models (PROC GLIMMIX, SAS 9.3)^29^ to account for the multilevel nature of the observational data, which consisted of monthly data points for physicians clustered within primary care clinics.

Physician-month was the unit of analysis, and main effects included both immediate (estimated by model intercepts) and incremental monthly effects (slopes) for three consecutive periods of analysis. These three periods included the first, second, and third years of Lean implementation in each clinic. We examined whether Lean primary care redesigns were associated with daily time savings during the course of physician workdays. To assess this, main effects of average work time immediately following the first year “post” Lean implementation were compared with baseline values of physician work times prior to Lean redesign. Subsequent values in the second and third years post-Lean implementation were compared to first-year results to assess whether initial effects of Lean redesigns were sustained over time.

We adjusted for secular trends and potential confounders in this non-randomized observational study, including physician scheduled clinic hours, mean age of patients on a physician’s panel, physician demographics, physician workload (e.g., number of office visits, telephone messages, patient emails), productivity (e.g., average number of RVUs per visit), and proportion of new patient visits. With physician work time as the outcome of interest, we also accounted for any interactions detected between a given post-Lean year and both physician workload and productivity measures for that year. Effects of Lean implementation on physician work hours were allowed to vary across clinics and thus included in the model as random intercepts.

## RESULTS

As shown in Table 1, physicians in our study sample spent a baseline average of 9 h working in the EHR each day, including at their desk, in exam rooms while seeing patients, and remotely offsite. Of this total amount of daily work time, they spent an average 6 h and 48 min with patients during office visits, and another 2 h and 10 min on desktop activities. Following the last patient visit of each day, physicians spent nearly 1.5 h still working after clinic hours. Among physicians who accessed the EHR remotely, approximately 30 min on average was spent working offsite daily. The bulk of this remote work time, approximately 27 min on average, occurred after the last patient visit each day.

**Table 1:** Sample Characteristics (*N* = 46 Primary Care Departments in 17 Clinical Facilities, 317 Physicians)

	Mean (or *N*)	SD (or %)	Min	Max
Primary care department				
Internal medicine	(15)	(32.6%)	–	–
Family medicine	(16)	(34.8%)	–	–
Pediatrics	(15)	(32.6%)	–	–
Practice size (FTE)	19.7	2.65	1.0	54.8
Staff:physician ratio	1.5	0.62	0	2.3
Study months post-Lean redesigns	43.6	1.41	36	57
Physician work time				
Total daily	9:02	1:33	4:18	13:35
Office visits	6:48	1:26	1:39	9:22
Desktop	2:10	1:01	0:30	5:22
After clinic (after last patient visit)	1:26	0:49	0:11	4:01
Remote work	0:30	0:33	0	2:25
Remote after clinic	0:27	0:30	0	2:11

*FTE*, full-time equivalent

Time units are in hours and minutes

Based on EHR access logs, Table 2 shows that in the first year after Lean implementation, physicians experienced an 8.3% or nearly 10-min decrease in the amount of time spent working *after* clinic hours each day, i.e., specifically after the last patient visit (95% CI: −13.8, −2.12). This reduction was sustained in the second year of Lean redesigns. By the third year, EHR access logs revealed a further 11.8% reduction in after-clinic work hours (95% CI: −23.5, −3.14) at the end of each day.

**Table 2:** After-Clinic Work Time (*N* = 317 Physicians)

	Percent change in work time	95% confidence interval
Baseline (pre-Lean)	–	–
1st year post-Lean	−8.3%†	−13.8, −2.12
2nd year post-Lean*	−0.8%‡	−5.44, 3.91
3rd year+ post-Lean**	−11.8%‡	−23.5, −3.14

This model based on observational EHR data is adjusted for physician-scheduled clinic hours, physician demographics, average age of patients on a physician’s panel, proportion of new patient visits, physician workload, productivity, and any interactions between post-Lean year, workload, and productivity

*Slope (monthly change) during 2nd year was −1.2% (95% CI: −2.13, −0.26)

† Reference: baseline (pre-Lean)

**Slope (monthly change) during 3rd year was −1.1% (95% CI: −2.04, −0.16)

‡ Reference: 1st year (post-Lean)

Table 3 shows results for physicians’ total amount of EHR desktop work time throughout the day, which includes after-clinic hours as reported above in Table 2. In the first year after Lean implementation, EHR logs revealed a 10.9% or 15-min daily reduction in total desktop activity among physicians (95% CI: −22.2, −2.03). By the third year of Lean redesigns, there was an additional 9.9% decrease (95% CI: −21.4, −0.11) totaling nearly a half hour saved in desktop EHR activity each day.

**Table 3:** Total Desktop Time (*N* = 317 Physicians)

	Percent change in work time	95% confidence interval
Baseline (pre-Lean)	–	–
1st year post-Lean	−10.9%†	−22.2, −2.03
2nd year post-Lean*	0.7%‡	−1.93, 5.05
3rd year+ post-Lean**	−9.9%‡	−21.4, −0.11

This model based on observatonal EHR data is adjusted for physician-scheduled clinic hours, physician demographics, average age of patients on a physician’s panel, proportion of new patient visits, physician workload, productivity, and any interactions between post-Lean year, workload, and productivity

*Slope (monthly change) during 2nd year was −1.2% (95% CI: −2.41, −0.24)

† Reference: baseline (pre-Lean)

**Slope (monthly change) during 3rd year was −1.0% (95% CI: −2.28, −0.16)

‡ Reference: 1st year (post-Lean)

Table 4 shows results for time spent providing direct care to patients during scheduled office visits. We found no immediate change in this measure during the first year of Lean redesigns. During the second year, an incremental decrease (downward slope) of 0.8% occurred reflecting several minutes of office time saved each month (95% CI: −1.41, −0.10). By the third year of Lean implementation, there was a more marked 18.6% reduction in office visit time, or approximately 1 h and 15 min, by the end of the study period as compared with the first year of Lean redesigns (95% CI: −29.3, −8.29).

**Table 4:** Office Visit Time (*N* = 317 Physicians)

	Percent change in work time	95% confidence interval
Baseline (pre-Lean)	–	–
1st year post-Lean	1.9%†	−3.66, 6.18
2nd year post-Lean*	0.7%‡	−2.47, 3.93
3rd year+ post-Lean	−18.6%‡	−29.3, −8.29

This model based on observational EHR data is adjusted for physician-scheduled clinic hours, physician demographics, average age of patients on a physician’s panel, proportion of new patient visits, physician workload, productivity, and any interactions between post-Lean year, workload, and productivity

*Slope (monthly change) during 2nd year was −0.8% (95% CI: −1.41, −0.10)

† Reference: baseline (pre-Lean)

‡ Reference: 1st year (post-Lean)

Table 5 presents results on the total amount of time that primary care physicians spent working each day. This measure included both desktop activity and time spent in office visits. Although no significant change was detected in the first year of Lean implementation, a gradual decrease of 0.8% per month occurred during the second year (95% CI: −1.23, −0.36) similar to the pattern observed with office visit time. This downtrend was followed in the third year by a 20% reduction in total daily work time (95% CI: −29.2, −9.60), yielding an average 1 h and 52 min saved in physician work by the end of the study period.

**Table 5:** Total Daily Work Time (*N* = 317 Physicians)

	Percent change in work time	95% confidence interval
Baseline (pre-Lean)	–	–
1st year post-Lean	−1.1%†	−3.87, 1.71
2nd year post-Lean*	0.9%‡	−1.53, 3.33
3rd year+ post-Lean	−20.0%‡	−29.2, −9.60

This model based on observational EHR data is adjusted for physician scheduled clinic hours, physician demographics, average age of patients on a physician’s panel, proportion of new patient visits, physician workload, productivity, and any interactions between post-Lean year, workload, and productivity

*Slope (monthly change) during 2nd year was −0.8% (95% CI: −1.23, −0.36)

† Reference: baseline (pre-Lean)

‡ Reference: 1st year (post-Lean)

We found no statistically significant associations between Lean redesigns and the number of hours that primary care physicians spent working remotely in the EHR. All statistically significant changes in practice were limited to the time when physicians were physically present onsite.

## DISCUSSION

This 6-year observational study of physician EHR access data found significant time savings associated with Lean primary care redesigns. In the first year of Lean implementation, physicians spent less time on EHR desktop tasks particularly after seeing their last patient each day. In the second year, EHR access logs showed gradual decreases in office visit time and closely related total work time. This finding corresponded with improvements in activities such as agenda setting, which aimed to focus patient time spent with physicians during office visits, and 5S standardization of exam room equipment to facilitate more seamless patient care. By the final year of Lean redesigns, the cumulative amount of daily time that physicians spent working each day decreased significantly.

Primary care physicians often work well beyond clinic hours, resulting in frequent logging of overtime and high potential for burnout. In a Lean approach to clinical workflows, physicians are encouraged to “batch” less by not allowing tasks to accumulate until the end of each day. Rather, they are encouraged to complete tasks using a “just-in-time” approach (i.e., continuously as needed between patient visits) such that incoming items are addressed in real time, thus reducing excessive amounts of unfinished work at the end of each day.

Consistent with this goal, an early objective for Lean redesigns in this study organization was the enabling of physicians to complete their work shortly after seeing the last patient each day. Based on related studies in primary care, physicians indicate that 15% of their total activities outside the exam room, with the exception of charting, could ideally be performed by clinical support staff. ^30^ Relevant activities include returning patient phone calls, replying to electronic messages, preparing prescription refill renewals, and coordinating care. By engaging team members where appropriate, Lean aims to utilize all available supports and resources to optimize care delivery. With each team member working up to scope, Lean redesigns also aim to achieve a more even distribution of labor where appropriate.^31,32^

In the study organization, new care team workflows and space redesigns were also implemented with a goal of streamlining care processes and increasing staff involvement in patient care. For example, joint care team management of the electronic inbox aimed to create a “just-in-time” flow of patient care tasks to be addressed by staff throughout the day. The goal was to reduce pile-up of items that physicians would typically need to address at the end of each day. With joint management of the inbox by non-physician care team members such as medical assistants, licensed vocational nurses, or registered nurses, both administrative and clinical care items were to be either directly addressed in real time or prepared for the physician’s immediate attention. This collaborative work was designed to achieve a more continuous and rapid completion of incoming tasks each day.^23^

Changes to physical workspace and care team roles also aimed to create time savings during patient visits. For example, “5S” standardization of exam rooms was designed to help physicians locate all needed supplies and educational materials at the point of care. “Agenda setting” by MAs at the start of each visit aimed to better structure patient-physician interactions while also making visits more predictable. In this new role, MAs helped prioritize patient concerns at the start of the appointment by asking a standard set of questions (e.g., “What are your top concerns for today’s visit?”) to focus time later spent with the physician. The intent was to have more planned discussions that would also result in a more timely cadence of start/end times for all appointments scheduled throughout the day.

Given these intended improvements, there are also known unintended consequences that have been cited in the Lean healthcare literature. Unanticipated results after Lean implementation in primary care include increased physician and staff burnout, workplace stress, and decreased patient satisfaction with some aspects of care immediately following Lean implementation.^15,22^ Based on systematic reviews conducted primarily in hospitals and emergency departments, other difficulties associated with Lean implementation include mixed results on staff satisfaction, work intensification, and inconsistent benefits to patient flow (see Table 6 for more information). Challenges may have stemmed from failures to successfully manage change or were a result of suboptimal approaches to introducing Lean redesigns. Nevertheless, further study on the outcomes of more effective Lean implementations is needed, ideally using more rigorous study designs.

**Table 6:** Potential Unanticipated Consequences of Lean

Results of studies found in systematic reviews^16–21^	
D’Andreamatteo A, et al. Lean in healthcare: A comprehensive review. Health policy. 2015;119(9), 1197–1209	• Mixed staff satisfaction and safety • Mixed improvements in support services (e.g., information technology)
Isfahani H, et al. Features and results of conducted studies using a Lean management approach in emergency department (ED) in hospitals: a systematic review. Bulletin of Emergency and Trauma. 2019;7(1):9–20	• Mismatch between job tasks, licensing constraints • Perception of being monitored • Work intensification • Lack of sustainability
Moraros J, et al. Lean interventions in healthcare: do they actually work? A systematic literature review. International Journal for Quality in Health Care. 2016;28(2), 150–165.	• No statistically significant association with patient satisfaction and health outcomes • Increased costs and worker dissatisfaction • Inconsistent benefits to patient flow and safety • Decreased nurse engagement, care quality, and patient safety
Zepeda-Lugo C, et al. Assessing the impact of Lean healthcare on inpatient care: a systematic review. International Journal of Environmental Research and Public Health. 2020;17(15), 5609–32.	• Mixed impact on length of stay • Mixed impact on readmission rates
Tlapa D, et al. Effects of Lean healthcare on patient flow: a systematic review. Value Health. 2020 Feb;23(2):260–273.	• Mixed length of stay, readmission rates • Mixed results for patients leaving ED without being seen
Souza D, et al. A systematic review on Lean applications in emergency departments. Healthcare*.* 2021; 9(6), 763–82.	• Increased tension between healthcare workers (Six Sigma was also used in this approach)

Our study findings have implications for work reallocation and resourcing in primary care. Early research in primary care found that nearly one-third to one-half of physicians’ workdays are spent on activities outside the exam room, predominately focused on documentation, performing tasks outside the exam room, and following up on patients not physically present.^30,33^ Another study in geriatrics found that 7 min of indirect patient care was provided outside the exam room for every 30 min of direct patient interaction.^34^ Based on the study’s calculations, this translated into 8 extra hours of time each week.

In light of these studies and others included in systematic reviews, the immediate implication for our findings is the clear need for additional work using alternative study designs (e.g., RCT) to strengthen and verify the impact of Lean redesigns in primary care. If Lean proves beneficial in this context, then there are important further implications for overstretched physicians who desire more autonomy in their work schedules. For example, with time released from administrative and routine clinical tasks, physicians would have the option of spending more quality time with patients, connecting with more patients by e-visit or phone, expanding clinic hours to accommodate new patients, or simply having shorter work days to mitigate burnout. Shifts in how physicians spend their time—particularly in moving from indirect to more direct patient care—align with Lean goals of maximizing value at work. Such goals may be achieved as physicians repurpose administrative or otherwise unbillable time for more value-added time providing direct patient care.

Although we did not focus on subjective reports of physician burnout, our study may be relevant to this long-standing problem in primary care. Job-related fatigue in medicine can negatively affect not only physician work experiences and performance, but also patient satisfaction with care. Factors contributing to experiences of burnout are excessive workloads and overburden by administrative work, regulatory policies, and a growing number of publicly reported performance metrics.^5^ Lean redesigns may help alleviate pressures by creating capacity among clinical staff and physicians to better address these requirements. This is another important area warranting further study.

Limitations to our analysis include lack of comparison groups as the health system comprehensively implemented Lean in all its primary care clinics. In the absence of an RCT, we adopted an interrupted time series approach to analyzing EHR-based observational data. This approach allowed each clinic to serve as its own control group based on baseline trends documented within their practice. We also applied a continuous employment criterion to ensure that physicians had been practicing in their clinic for a sufficient amount of time to attribute changes in their work patterns to the introduction of Lean redesigns. While this criterion posed a possibility that physicians simply became more efficient over time, our adjustment for productivity levels, secular trends, and examination of three consecutive post-Lean periods was designed to provide a more robust analysis of Lean impacts on physician work over time.

## CONCLUSION

Lean care transformations have been used to foster staff engagement, value-based work cultures, and physician well-being. Despite numerous studies on system-wide efficiency, none to date examined Lean impacts on daily practice patterns among primary care physicians and their time spent working each day. Our findings based on observational EHR data suggest that Lean can help capture a valuable resource in the form of time savings for physicians. While decreases were initially observed in time spent on desktop work and during after-clinic hours, in later years cumulative decreases were observed in both office visit time and total daily work time. Findings are suggestive of the potential for Lean redesigns to create work efficiencies, which may allow physicians more options in their medical practice while fostering a greater sense of professional autonomy.

## References

[1] Shanafelt TD, Hasan O, Dyrbye LN (2015). Changes in Burnout and Satisfaction With Work-Life Balance in Physicians and the General US Working Population Between 2011 and 2014. Mayo Clin Proc..

[2] Westbrook JI, Ampt A, Kearney L, Rob MI (2008). All in a day’s work: an observational study to quantify how and with whom doctors on hospital wards spend their time. Med J Aust..

[3] Sinsky C, Colligan L, Li L (2016). Allocation of physician time in ambulatory practice: a time and motion study in 4 specialties. Ann Intern Med..

[4] Arndt BG, Beasley JW, Watkinson MD (2017). Tethered to the EHR: Primary Care Physician Workload Assessment Using EHR Event Log Data and Time-Motion Observations. Ann Fam Med..

[5] Dyrbye LN, Shanafelt TD (2011). Physician burnout: A potential threat to successful health care reform. JAMA..

[6] Wallace JE, Lemaire JB, Ghali WA (2009). Physician wellness: a missing quality indicator. The Lancet..

[7] Shanafelt TD, Dyrbye LN, West CP (2017). Addressing Physician Burnout: The Way Forward. JAMA..

[8] Shanafelt TD, Boone S, Tan L (2012). Burnout and satisfaction with work-life balance among us physicians relative to the general us population. Arch Intern Med..

[9] Shah R, Ward PT (2007). Defining and developing measures of lean production. J Oper Manag..

[10] Spear S, Bowen HK (1999). Decoding the DNA of the Toyota production system. Harv Bus Rev..

[11] Womack JP, Byrne AP, Flume OJ, Kaplan GS, Toussaint J (2005). *Going Lean in Health Care*.

[12] Jimmerson C, Weber D, Sobek DK (2005). Reducing waste and errors: piloting lean principles at Intermountain Healthcare. Jt Comm J Qual Patient Saf..

[13] Kim CS, Spahlinger DA, Billi JE (2009). Creating value in health care: the case for Lean thinking. JCOM..

[14] Sullivan P, Soefje S, Reinhart D, McGeary C, Cabie ED (2014). Using Lean methodology to improve productivity in a hospital oncology pharmacy. Am J Health Syst Pharm..

[15] Hung DY, Harrison MI, Martinez MC, Luft HS (2017). Scaling Lean in Primary Care: Impacts on System Performance. Am J Manag Care..

[16] D’Andreamatteo A, Ianni L, Lega F, Sargiacomo M (2015). Lean in healthcare: A comprehensive review. Health policy..

[17] Isfahani H, Tourani S, Seyedin H (2019). Features and results of conducted studies using a Lean management approach in emergency department in hospitals: a systematic review. Bulletin of Emergency and Trauma..

[18] Moraros J, Lemstra M, Nwankwo C (2016). Lean interventions in healthcare: do they actually work? A systematic literature review. International Journal for Quality in Health Care..

[19] Zepeda-Lugo C, Tlapa D, Baez-Lopez Y, Limon-Romero J, Ontiveros S, Perez-Sanchez A, Tortorella G (2020). Assessing the impact of Lean healthcare on inpatient care: a systematic review. International Journal of Environmental Research and Public Health..

[20] Tlapa D, Zepeda-Lugo CA, Tortorella GL, Baez-Lopez YA, Limon-Romero J, Alvarado-Iniesta A, Rodriguez-Borbon MI (2020). Effects of Lean healthcare on patient flow: a systematic review. Value Health..

[21] Souza DL, Korzenowski AL, Alvarado MM, Sperafico JH, Ackermann A, Mareth T, Scavarda AJ (2021). A systematic review on Lean applications in emergency departments. Healthcare..

[22] Hung DY, Harrison MI, Truong Q, Du X (2018). Experiences of primary care physicians and staff following lean workflow redesign. BMC Health Serv Res..

[23] Hung DY, Truong Q, Liang SY (2021). Implementing Lean Quality Improvement in Primary Care: Impact on Efficiency in Performing Common Clinical Tasks. J Gen Intern Med..

[24] Wagner AK, Soumerai SB, Zhang F, Ross-Degnan D (2002). Segmented regression analysis of interrupted time series studies in medication use research. J Clin Pharm Ther..

[25] Gebski V, Ellingson K, Edwards J, Jernigan J, Kleinbaum D (2012). Modelling interrupted time series to evaluate prevention and control of infection in healthcare. Epidemiol Infect..

[26] Penfold RB, Zhang F (2013). Use of interrupted time series analysis in evaluating health care quality improvements. Acad Pediatr..

[27] Hu Y, Richards DA, Hallsberg I (2015). Stepped wedge, natural experiments and interrupted time series analysis designs. *Complex Interventions in Health: An Overview of Research Methods*.

[28] **Highfield L**, **Rajan SS**, **Valerio MA**, **Fernandez ME**, **Bartholomew LK**. A non-randomized controlled stepped wedge trial to evaluate the effectiveness of a multi-level mammography intervention in improving appointment adherence in underserved women. *Implement Sci.* 2015;10(143). 10.1186/s13012-015-0334-x PMC4604615 26464110

[29] *The GLIMMIX Procedure: PROC GLIMMIX* [computer program]. Version 9.3. Cary, NC.

[30] Chen M, Hollenberg J, Michelen W, Peterson J, Casalino L (2011). Patient Care Outside of Office Visits: A Primary Care Physician Time Study. J Gen Intern Med..

[31] Willard-Grace R, Hessler D, Rogers E, Dubé K, Bodenheimer T, Grumbach K (2014). Team Structure and Culture Are Associated With Lower Burnout in Primary Care. The Journal of the American Board of Family Medicine..

[32] Dunn PM, Arnetz BB, Christensen JF, Homer L (2007). Meeting the imperative to improve physician well-being: assessment of an innovative program. J Gen Intern Med..

[33] Gottschalk A, Flocke SA (2005). Time spent in face-to-face patient care and work outside the examination room. Ann Fam Med..

[34] Farber J, Siu A, Bloom P (2007). How much time do physicians spend providing care outside of office visits?. Ann Intern Med..

